# Impaired Treg‐Mediated Immune Regulation in Peri‐Implantitis Lesions and Implant Loss: Insights From Histological and Molecular Analyses

**DOI:** 10.1111/jcpe.70026

**Published:** 2025-08-26

**Authors:** Emilio A. Cafferata, Ausra Ramanauskaite, Puria Parvini, Clemens Raabe, Eva Dohle, Shahram Ghanaati, Frank Schwarz

**Affiliations:** ^1^ Department of Oral Surgery and Implantology Goethe University Frankfurt am Main Germany; ^2^ Oral Peri‐Implant Research Group, School of Dentistry Universidad Científica del Sur Lima Peru; ^3^ Department of Oral Surgery and Stomatology, School of Dental Medicine University of Bern Bern Switzerland; ^4^ FORM, Frankfurt Orofacial Regenerative Medicine, Department for Oral, Cranio‐Maxillofacial and Facial Plastic Surgery Medical Center of the Johann Wolfgang Goethe University Frankfurt Germany

**Keywords:** crevicular fluid, granulation tissue, inflammation, peri‐implantitis, T regulatory cells

## Abstract

**Aim:**

To evaluate the T regulatory lymphocyte (Treg) profile and its potential contribution to peri‐implant tissue destruction during peri‐implantitis (PI).

**Methods:**

PI granulation tissue and crevicular fluid collected during PI surgical (PI group, *n* = 23) and explantation (PI‐X group, *n* = 23) therapy, with peri‐implant healthy tissue from second‐stage surgery (H group, *n* = 20) as controls, were analysed. The inflammatory infiltrate was characterised by H&E staining. The relative expression of Treg‐associated transcription factors and cytokines was assessed by RT‐qPCR. Forkhead box P3 (FOXP3) and neuropilin (NPR)‐1 were detected by immunohistochemistry, and interleukin (IL)‐10, TGF‐β1 and IL‐35 by ELISA. The clinical parameters, namely probing depth (PD), bleeding on probing (BOP) and vertical defect depth (VDD), were also recorded.

**Results:**

PI and PI‐X lesions showed up‐regulation of *FOXP3*, *HELIOS* and *IL35B* and down‐regulation of *NRP1* and *TGFβ1* mRNA expression, compared to H tissue (*p* < 0.05). Significantly more FOXP3^+^ cells and significantly less NRP‐1^+^ area were detected in PI and PI‐X lesions (*p* < 0.05). IL‐35 levels were up‐regulated, whereas TGF‐β1 levels were down‐regulated in PI and PI‐X lesions, compared to H samples (*p* < 0.05). PD and VDD were significantly correlated with the down‐regulation of FOXP3 and NRP‐1 (*p* < 0.05).

**Conclusions:**

Treg dysfunction and altered cytokine profiles in PI are associated with inflammation and clinical disease severity.

## 1 Introduction

Peri‐implantitis (PI), characterised by non‐resolving inflammation accompanied by progressive bone loss, is one of the leading causes of late implant loss (Zhai et al. 2024). Although different treatments exist, achieving complete disease resolution—as defined by the EFP guidelines—remains challenging (Monje and Nart 2024; Ramanauskaite et al. 2025). Nonetheless, surgical therapy can result in significant improvements, such as reduced probing depths and stabilised or even improved marginal bone levels (La Monaca et al. 2025; Schwarz et al. 2018). Even though resolution is achieved in ⁓60% of cases over 5–10 years, PI recurrence requiring retreatment or leading to implant loss might be expected, especially in advanced lesions or in patients with unfavourable risk profiles (Isler et al. 2024; Ramanauskaite et al. 2025). Therefore, while surgical treatment can be effective in managing PI, its long‐term success may be influenced by factors beyond bacterial control, including host immune regulation—a component that remains insufficiently understood (Yin et al. 2024).

In this context, an intricate sequence of immune responses dictates the fate of dental implant integration and maintenance (Davies 2003; Insua et al. 2017). However, when this finely tuned equilibrium is lost—for example, through microbial dysbiosis—the resulting dysregulated immune response may lead to inflammatory osteolysis, such as those occurring during PI and periodontitis (Malmqvist et al. 2024). Similarly, periodontitis is also characterised by inflammation‐driven alveolar bone loss, which eventually can lead to tooth loss. However, PI lesions present larger areas of immune cell infiltration and bone resorption than periodontitis (Berglundh et al. 2011; Blanco et al. 2021). In this context, the immunopathogenesis of periodontitis partly relies on the deregulation of the immunoregulatory axis of the immune response mediated by T regulatory cells (Tregs). Indeed, Tregs, characterised by the expression of the transcription factor forkhead box p3 (FOXP3) and the interleukin (IL)‐2 receptor, cluster of differentiation (CD)25, modulate innate and adaptive immunity and maintain the oral immunological homeostasis, which is essential for periodontal health (Alvarez et al. 2018).

Although during periodontitis the periodontal tissue enriched in pro‐inflammatory mediators may induce a Treg dysfunctional phenotype that promotes alveolar bone destruction (Alvarez et al. 2020), nonetheless, in the case of PI, the role of these cells has not been described yet.

Therefore, the aim of this study was to compare Tregs′ presence and Treg‐associated marker expression between clinically healthy peri‐implant tissues and PI lesions from different clinical scenarios (i.e., needing surgical reconstructive therapy and indicated for explantation), in order to identify immunoregulatory profiles linked to PI disease severity.

## 2 Materials and Methods

### 2.1 Study Design

The present study analysed the clinical records and matching preserved peri‐implant tissue samples from patients treated between 2022 and 2024 at the Department of Oral Surgery and Implantology, Carolinum, Goethe University Frankfurt. In accordance with the protocol (N:2022–652), approved by the local ethics committee, the study was designated as retrospective, as it involved previously collected clinical and biological material. Details of the methods are available in Supporting Information.

### 2.2 Participant Selection

A total of 66 patient samples were included: 46 had a clinical diagnosis of PI, of whom 23 (*n* = 23 implants) underwent PI surgical therapy (PI group) and 23 (*n* = 23 implants) underwent explantation (PI‐X group). As a control, 20 healthy implant samples (i.e., without signs of progressive bone loss and no bleeding on probing) (PI‐H group) were used (Table S1).

Inclusion criteria: Implants with complete clinical records and matching tissue samples, indicating > 2 mm keratinised mucosa and plaque index < 1 (Löe 1967), were included. For PI‐implants, inclusion required PI diagnosed as follows: bleeding on probing (BOP) and/or suppuration (SUPP); probing depth (PD) ≥ 6 mm; and radiographic marginal bone loss (MBL) ≥ 3 mm (Berglundh et al. 2018). For PI‐X, explantation was indicated when PI‐affected implants exhibited radiographic bone loss of more than or equal to two‐thirds of the implant length and/or mobility. For H, inclusion required no BOP/SUPP, PD ≤ 5 mm and no bone loss beyond remodelling. Clinical parameters were recorded at six sites per implant, which included BOP, PD and vertical defect depth (VDD)—measured intra‐surgically from the bone crest to the deepest point of the intrabony defect using a periodontal probe. For the H group, clinical parameters were taken 1–3 months after loading.

Exclusion criteria: Patients with inflammatory disorders, recent antibiotic or immunosuppressant therapy, pregnancy/lactation and untreated periodontitis were excluded to minimise systemic and local inflammatory confounding effects on peri‐implant tissue immune profiles (Ginesin et al. 2023; Roccuzzo et al. 2012).

### 2.3 Peri‐Implantitis Reconstructive Surgical Procedure and Peri‐Implant Tissue Sample Obtention

Before surgery, all PI‐affected implants, except in the PI‐X group, received pre‐operative supra‐mucosal implant cleaning and were treated through a standardised surgical protocol S1 (Schwarz et al. 2017).

In the PI and PI‐X groups, granulation tissue was carefully curetted directly from the implant surface and defect area. In the H group, soft‐tissue biopsy samples were collected from the mucosa directly overlying the implant cover screw using a circular biopsy punch during the second stage of implant surgery. One biopsy sample was collected per patient and divided, using a sterile blade, into two portions: a small fragment (~2–5 mg) for RNA extraction and the rest for immunohistochemistry.

### 2.4 Immunohistochemistry

Because of the irregular—for instance, lacking distinct epithelial landmarks—and often fragmented morphology of granulation tissue from PI and PI‐X samples, histological orientation was not feasible. In contrast, H samples—with regular tissue architecture—were oriented by embedding them with the implant‐contacting surface facing downwards in the mould. This positioning allowed the obtention of coronal sections with the epithelial surface aligned towards the external mucosal interface. Formalin‐fixed, paraffin‐embedded sections were stained with anti‐FOXP3 and anti‐neuropilin (NRP)‐1 antibodies. Detection employed 3,3′‐diaminobenzidine (DAB) or 3‐amino‐9‐ethylcarbazole (AEC) chromogens and Mayer's haematoxylin counterstaining. Negative controls included IgG isotype and omission of primary antibodies.

### 2.5 Immunohistochemical Data Analysis

Digital images (×100) were analysed using ImageJ. For each sample, four non‐overlapping high‐power fields with well‐preserved morphology were randomly selected to define the regions of interest (ROIs). FOXP3^+^ cells were quantified as a percentage of total nuclei after grayscale conversion and thresholding, while NRP‐1^+^ staining was calculated as the percentage of positively stained membrane/cytoplasmic area within the ROI after background correction, as previously described (Cafferata et al. 2024; Ling et al. 2018).

### 2.6 RT‐qPCR Analysis

Total RNA was extracted using TRIzol (Cafferata et al. 2020). Then, cDNA was synthesised using a reverse transcription kit and amplified using the specific primers—*FOXP3*, *HELIOS*, *NRP1*, *IL10*, *TGFB1* and *IL35B* (Table S2)—and a qPCR kit. 18S rRNA expression levels were used as an endogenous control.

### 2.7 Peri‐Implant Crevicular Fluid Sample Analysis

To complement peri‐implant tissue analyses, peri‐implant crevicular fluid samples—representing the extracellular inflammatory context—collected prior to surgery in the PI and PI‐X groups, and during follow‐up in the H group, were analysed. For these analyses, the target site was gently dried and isolated with cotton rolls and, if present, supragingival plaque was removed using a plastic curette while avoiding the marginal mucosa. Then, two paper strips were inserted into the mesial and distal sites of the peri‐implant sulcus of the implant for 30 s (Navarrete et al. 2023). The levels of IL‐10, TGF‐β1 and IL‐35 were analysed by ELISA.

### 2.8 Data Analysis

Data were expressed as mean ± standard deviation and analysed in a cross‐sectional manner. Normality was assessed with the Kolmogorov–Smirnov test. Inter‐group comparisons used the Kruskal–Wallis and Mann–Whitney U tests. Correlations were assessed via Spearman's rank test. Multiple linear and logistic regressions were performed to evaluate associations between Treg‐marker expression and clinical parameters, adjusting for age and smoking. A sample size of *n* = 20 per group ensured 80% power to detect group differences (effect size *f* = 0.65, *α* = 0.05). Statistical analyses were performed using Jamovi v2.3 and Excel, with significance set at *p* < 0.05.

## 3 Results

### 3.1 Histological Depiction of Healthy Peri‐Implant Tissue and Peri‐Implantitis‐Affected Tissue

**FIGURE 1:**
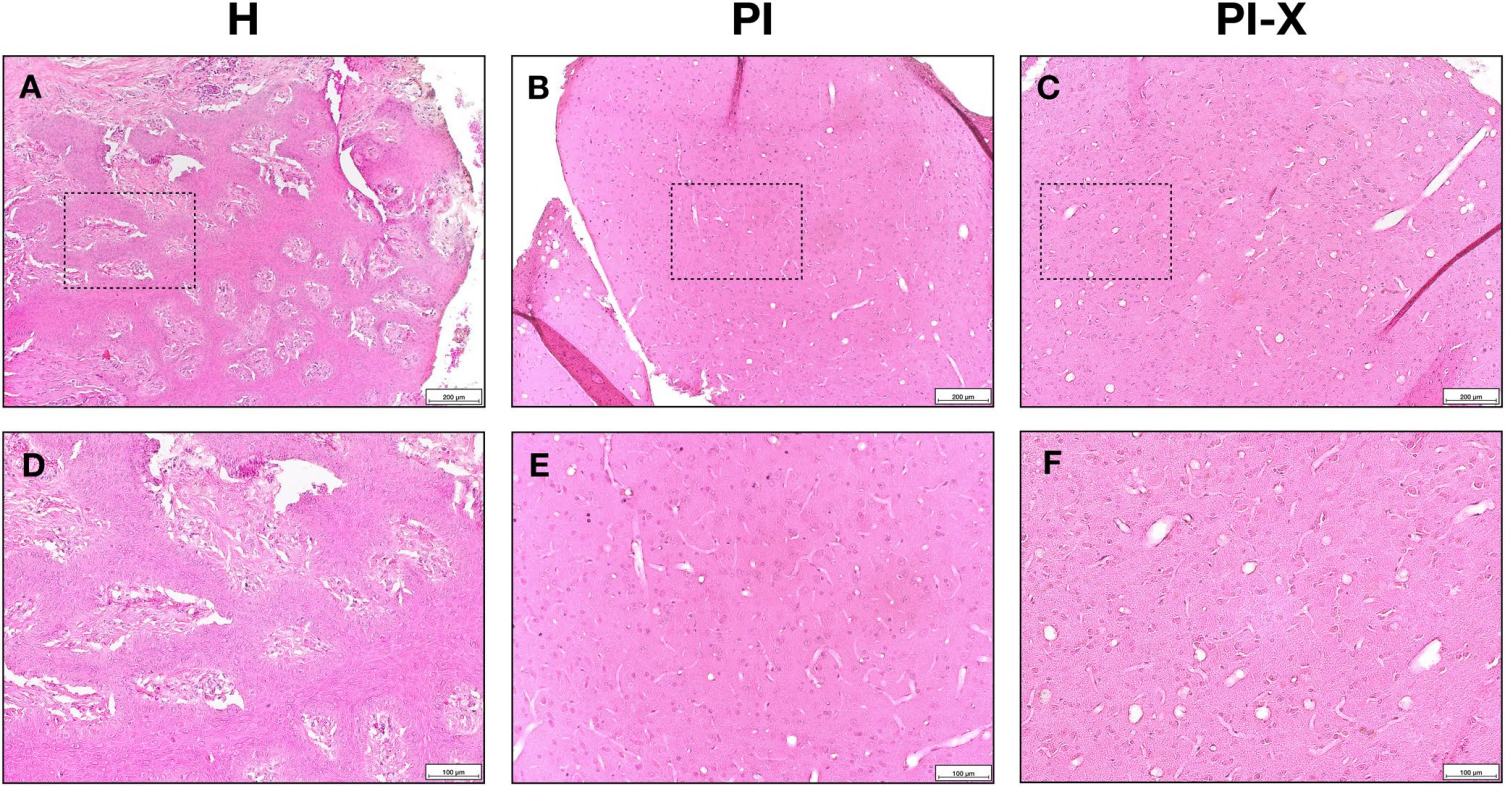
Histological comparison of healthy peri‐implant tissue and peri‐implantitis‐affected tissue. Representative haematoxylin and eosin (H&E)‐stained tissue sections from each study group are shown. Upper panels at (×10) magnification show (A) healthy peri‐implant tissue (H group), (B) granulation tissues obtained during peri‐implantitis surgical therapy (PI) and (C) explantation (PI‐X). Scale bar: 200 μm. Lower panels show (×20) magnification of the same sections: (D) H tissue, (E) PI tissue and (F) PI‐X tissue. Scale bar: 100 μm.

Samples from the H group showed no signs of epithelium disruption next to densely arranged collagen fibres within the connective tissue, and minimal presence of immune cells (Figure 1A–D). Samples from the PI and PI‐X groups exhibited the presence of fragmented and disorganised collagen bundles, along with numerous small capillaries accompanied by inflammatory cells, predominantly lymphocytes and macrophage‐like cells (Figure 1B–E,C–F). No evident morphological differences could be seen between the H&E‐stained slides of PI and PI‐X groups.

### 3.2 Tregs Phenotype–Associated Transcription Factors mRNA Expression in Peri‐Implant Tissues

**FIGURE 2:**
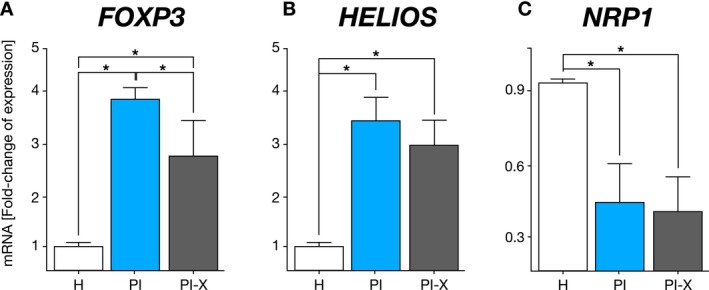
Expression of T‐regulatory cells–associated transcription factors. (A) *FOXP3*, (B) *HELIOS* and (C) *NRP1* mRNA expression levels in the healthy peri‐implant tissue, and granulation tissue from peri‐implantitis lesions. As reference for fold‐change in expression, the transcription factor mRNA expression in peri‐implant healthy (H) tissue was considered as 1. Data are represented as mRNA fold‐change and shown as mean ± SD (*n* = 20). Each experiment was performed in triplicate. *p < 0.05. H, healthy peri‐implant tissue group; PI, peri‐implantitis group; PI‐X, peri‐implantitis explanted implant group.

*FOXP3*, *HELIOS* and *NRP1* mRNA—encoding FOXP3, Helios and NRP‐1, respectively—expression levels, associated with the Tregs cell phenotype, were analysed (Figure 2). *FOXP3* expression was significantly up‐regulated in the PI and PI‐X groups, in comparison with H tissues. Specifically, *FOXP3* mRNA levels showed a mean fold‐change increase of 3.8 (*p* < 0.001) in the PI group, indicating an active Tregs response in the presence of peri‐implant inflammation. However, the PI‐X group showed a marked down‐regulation of *FOXP3*, with expression levels reduced by approximately 1.1‐fold relative to the PI group (*p* = 0.02) (Figure 2A). Similarly, the relative expression levels of *HELIOS* were significantly up‐regulated in the PI and PI‐X groups, in comparison with H controls (*p* < 0.001) (Figure 2B). In contrast to *FOXP3* and *HELIOS*, *NRP1* mRNA expression was consistently down‐regulated in both PI and PI‐X groups, in comparison with H controls (*p* = 0.04) (Figure 2C).

### 3.3 FOXP3 ^+^ Tregs and NRP‐1^+^ Cell Detection in Peri‐Implant Tissues

**FIGURE 3:**
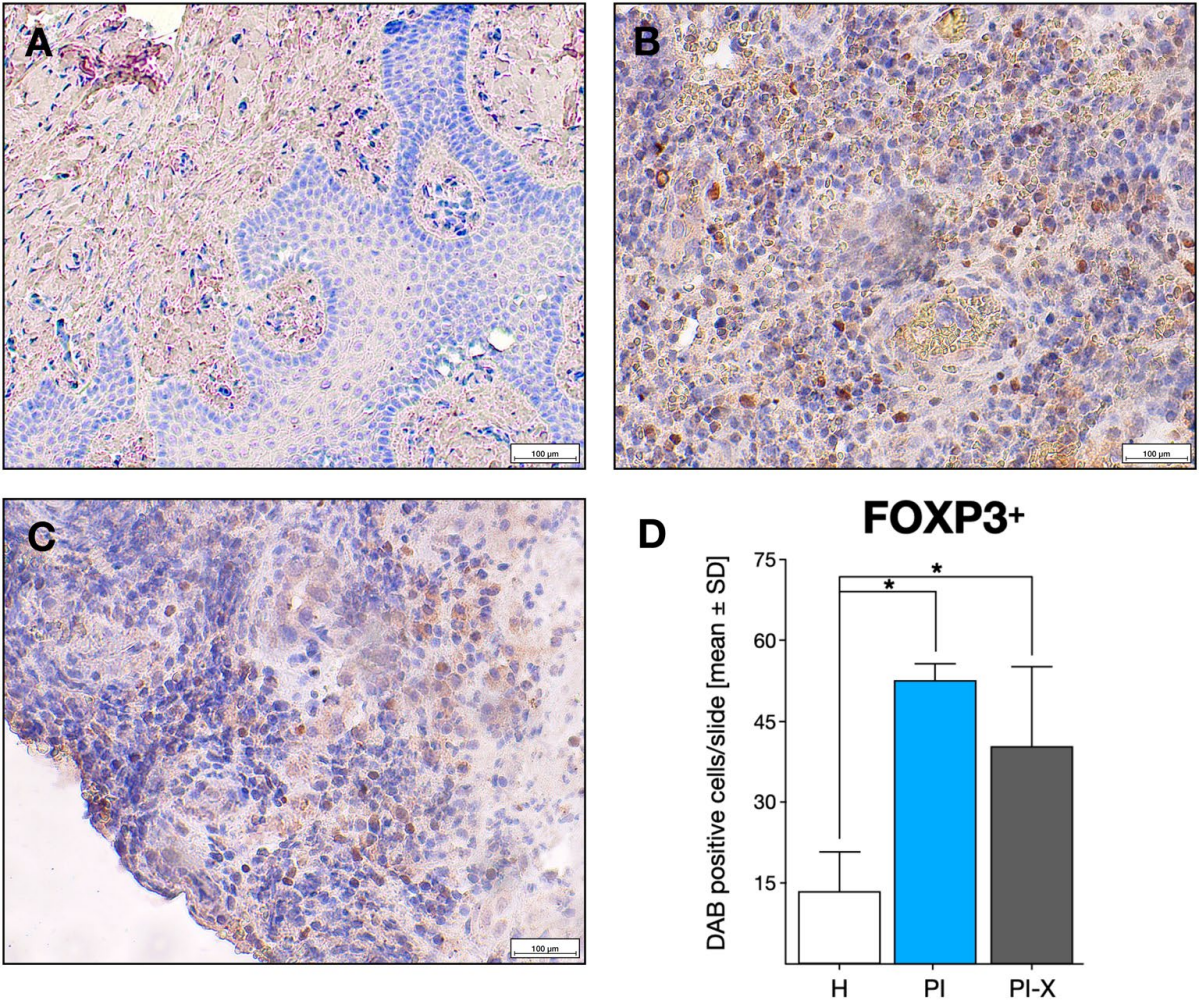
Immunostaining of FOXP3^+^ T regulatory cells. Representative images of the (A) healthy peri‐implant tissue group, (B) peri‐implantitis group, (C) peri‐implantitis explanted implants group showing FOXP3^+^ cells at (×20) augmentation. (D) Relative quantification of FOXP3^+^ cells in the peri‐implant tissues of the H, PI and PI‐X groups. Data are represented as mean ± SD positive cells from 10 random slides per sample (*n* = 20). *p < 0.05. H, healthy peri‐implant tissue group; PI, peri‐implantitis group; PI‐X, Peri‐implantitis explanted implants group. Scale bar: 100 μm.

In order to confirm the presence of Tregs in peri‐implant tissue, FOXP3 and NRP‐1 markers′ immunohistochemical analysis was performed (Figure 3). A significantly higher number of FOXP3^+^ Tregs cells were detected in PI and PI‐X samples compared with H tissues (*p* < 0.001) (Figure 3C). There was no significant difference between FOXP3^+^ cell numbers when comparing samples from PI and PI‐X (*p* = 0.077). Notably, in two samples from the H and the PI‐X groups, no FOXP3 immuno‐positive signal was observed.

**FIGURE 4:**
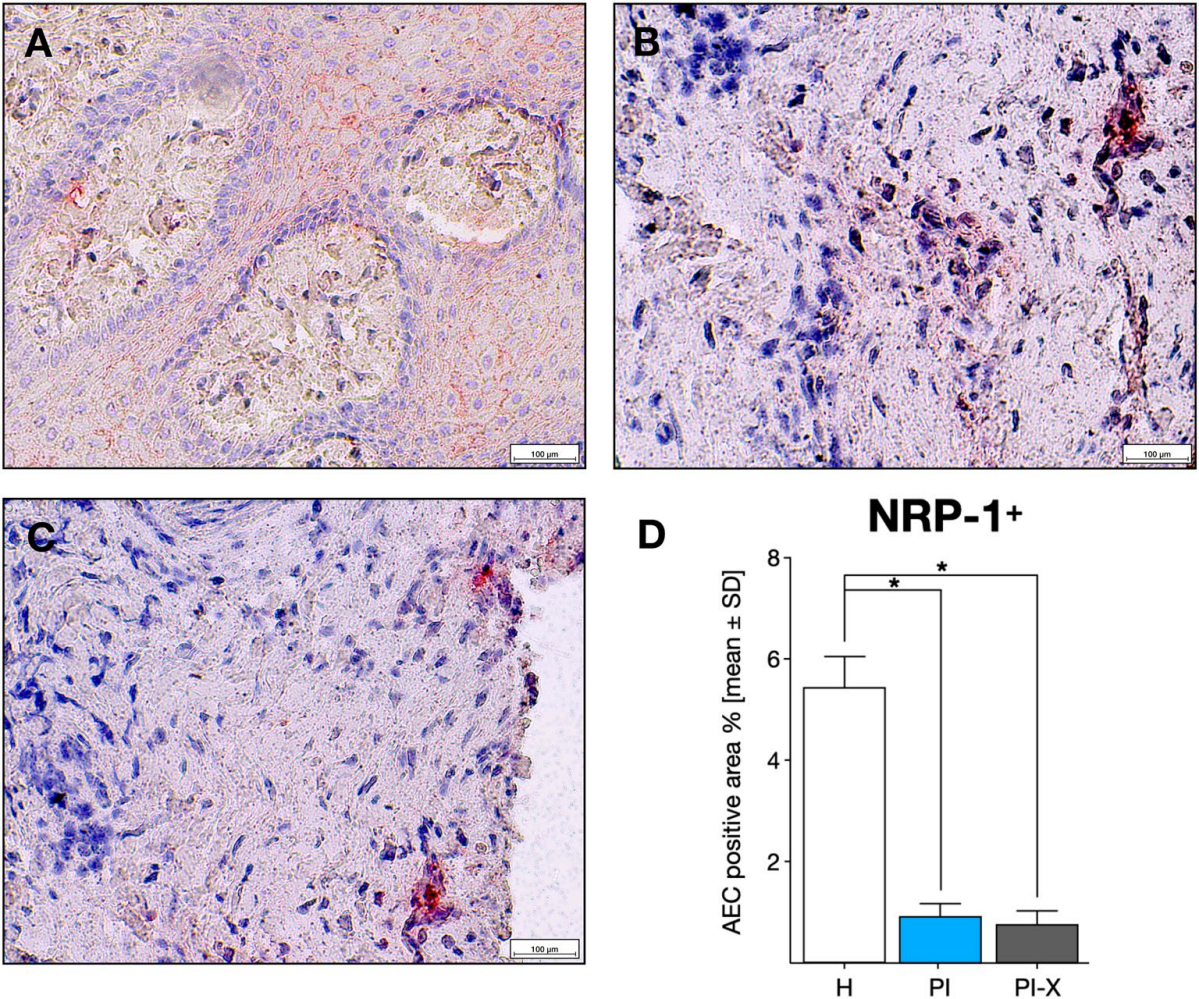
Immunostaining of NRP‐1^+^ cells. Representative images of the (A) healthy peri‐implant tissue group, (B) peri‐implantitis group, (C) peri‐implantitis explanted implants group showing NRP‐1^+^ cells at (×20) augmentation. (D) Relative quantification of NRP‐1^+^ cells in the peri‐implant tissues of the H, PI and PI‐X groups. Data are represented as mean ± SD positive cells from 10 random slides per sample (*n* = 20). *p < 0.05. H, healthy peri‐implant tissue group; PI, peri‐implantitis group; PI‐X, peri‐implantitis explanted implants group. Scale bar: 100 μm.

For NRP‐1, the positive area was significantly larger in H tissue samples than in PI and PI‐X group samples (*p* < 0.001) (Figure 4C). In healthy tissues, NRP‐1^+^ staining was predominantly observed at the cellular membrane of epithelial cells (Figure 4A). In contrast, in PI‐affected tissues, the infrequent NRP‐1^+^ staining was primarily localised at the cytoplasm of mostly macrophage‐like cells, followed by lymphocytes (Figure 4B). Two samples from the PI and PI‐X groups had no observable positive NRP‐1 immunodetection.

### 3.4 Treg Cytokine mRNA Expression in Peri‐Implant Tissues

**FIGURE 5:**
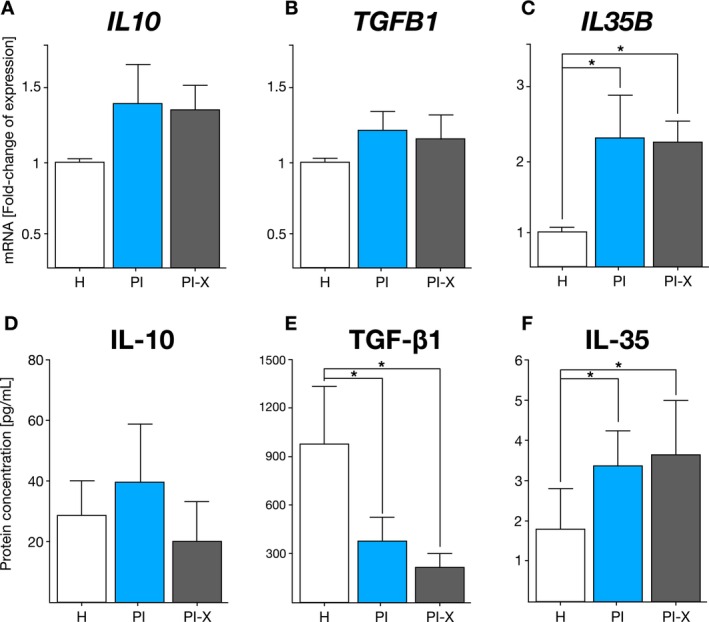
T regulatory cells‐associated cytokine expression and production. (A) *IL10*, (B) *TGFB1* and (C) *IL35B* mRNA expression in the healthy peri‐implant tissue, and granulation tissue from peri‐implantitis lesions. As reference for fold‐change in expression, the cytokine mRNA expression in the peri‐implant healthy (H) tissue was considered as 1. Data are represented as mRNA fold‐change and shown as mean ± SD (*n* = 20). Each experiment was performed in triplicate. (C) IL‐10, (D) TGF‐β1 and (E) IL‐35 protein levels in the peri‐implant crevicular fluid from the healthy implants, and peri‐implantitis‐affected implants. Data are represented as pg/mL and shown as mean ± SD (*n* = 20). Each experiment was performed in duplicate. *p < 0.05. H, healthy peri‐implant tissue group; PI, peri‐implantitis group; PI‐X, peri‐implantitis explanted implants group.

The mRNA expression levels of *IL10*, *TGFB1* and *IL35B*—encoding IL‐10, TGF‐β1 and IL‐35, respectively—associated with Treg‐mediated cytokine immune regulation were analysed (Figure 5). *IL10* mRNA relative expression showed no significant differences between healthy (H) and PI‐affected (PI and PI‐X) tissues (*p* = 0.052) (Figure 5A). Similarly, *TGFB1* mRNA levels were comparable across H, PI and PI‐X groups (*p* = 0.120) (Figure 5B). In contrast, *IL35B* mRNA expression was significantly up‐regulated in PI and PI‐X samples, showing a mean 2.3‐fold increase, in comparison with healthy (H) tissues (*p* = 0.026) (Figure 5C).

### 3.5 Tregs Cytokine Protein Levels in Peri‐Implant Crevicular Fluid

Subsequently, the protein levels of IL‐10, TGF‐β1 and IL‐35 were measured in the peri‐implant crevicular fluid. IL‐10 protein levels remained relatively unaltered across all groups (*p* = 0.11) (Figure 5D); however, TGF‐β1 protein levels were significantly lower in the PI and PI‐X groups, in comparison with H samples (*p* < 0.001) (Figure 5E). Consistent with the mRNA expression results, IL‐35 protein levels were significantly higher in both the PI and PI‐X groups compared with H (*p* = 0.004), while no significant difference was detected between PI and PI‐X (*p* = 0.15) (Figure 5F).

### 3.6 Tregs Activity and Clinical Signs of Peri‐Implantitis Association

**FIGURE 6:**
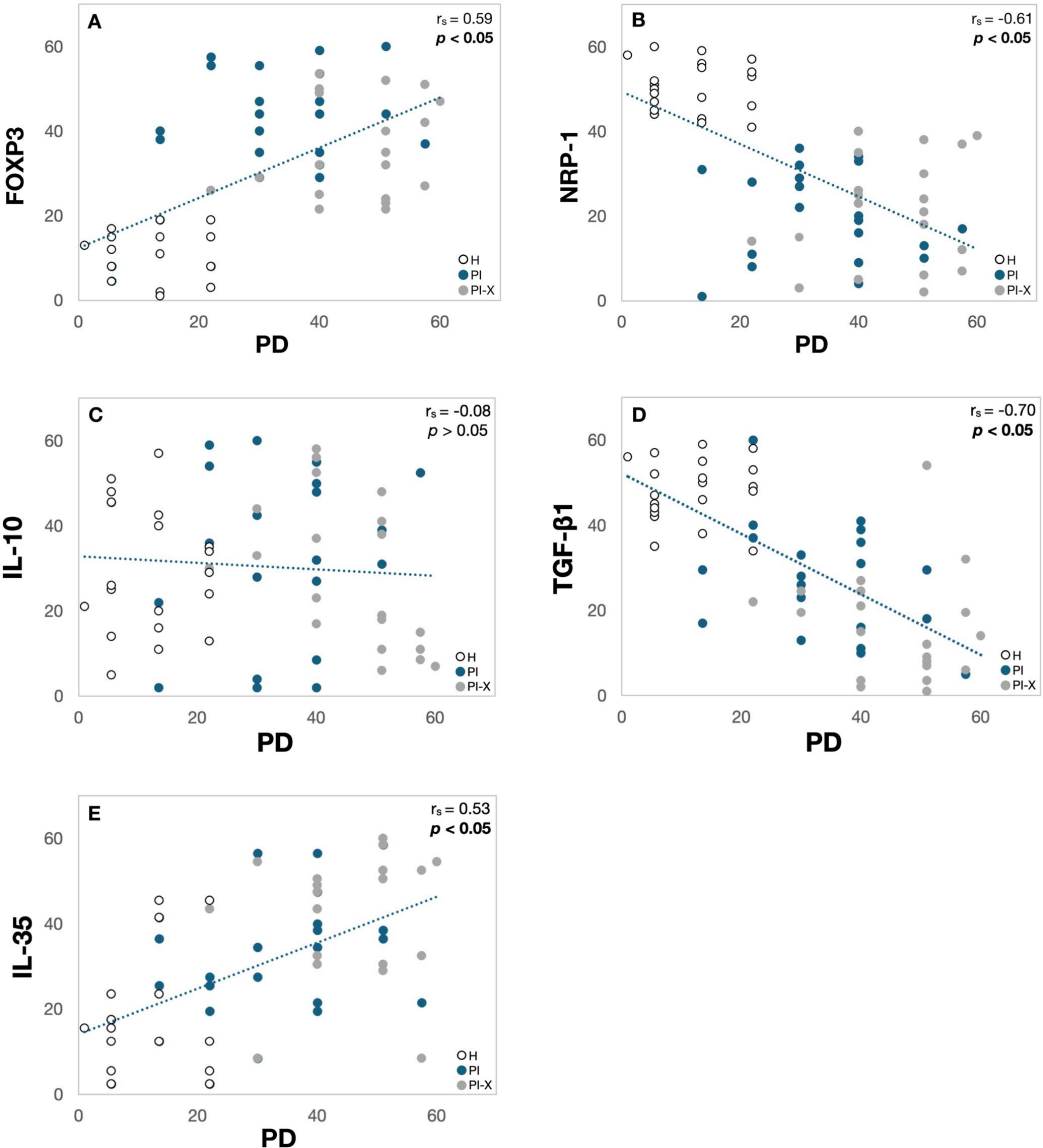
T regulatory cell–associated protein levels and probing depth correlation analysis. Scatter plots showing the correlation between ranked protein levels and probing depth in peri‐implant tissues. (A) FOXP3 vs. PD, (B) NRP1 vs. PD, (C) IL‐10 vs. PD, (D) TGF‐β1 vs. PD and (E) IL‐35 vs. PD. Spearman's rank correlation coefficient (*r*_s_) and the corresponding *p*‐values are displayed for each analysis. All implants were considered for analysis (PI = 23, PI‐X = 23, H = 20). Dots represent PD rank plotted against mean protein levels for a specific sample: White: OH, Blue: PI, Grey: PI‐X. Statistically significant correlations are depicted in bold.

FOXP3 expression was significantly associated with PD (*r*_s_ = 0.59, *p* < 0.001), BOP (OR = 1.17[0.01], *p* < 0.001) and VDD (0.04[0.01], *p* = 0.01) (Figure 6A; Tables 1A and 1B). On the contrary, NRP‐1 expression exhibited a significant inverse correlation with PD (*r*_*s*_ = −0.61, *p* < 0.001) and negative associations with BOP (OR = 0.20[0.38], *p* = 0.00002) and VDD (−0.40[0.13], *p* < 0.001) (Figure 6B; Tables 1A and 1B), suggesting that the increased presence of FOXP3^+^ Treg cells and decreased NRP‐1 expression are associated with increased peri‐implant tissue destruction.

**TABLE 1A:** Multiple logistic regression analysis for bleeding on probing as dependent variable with FOXP3, NRP‐1, IL‐10, TGF‐β1 and IL‐35 protein levels.

For BOP	OR (SE)	95% CI	*p*
FOXP3	1.17 (0.01)	[0.14; 0.18]	< 0.05*
NRP‐1	0.20 (0.38)	[−2.38; −0.89]	< 0.05*
IL‐10	0.99 (0.09)	[−0.19; 0.17]	0.91
TGF‐β1	1.00 (0.00)	[−0.01; 0.00]	< 0.05*
IL‐35	1.86 (0.10)	[0.42; 0.82]	< 0.05*

*Note*: All implants were considered for analysis (PI = 23, PI‐X = 23, H = 20). The regression models were adjusted for age and smoking status as covariates. BOP: bleeding on probing; OR: odds ratio; SE: standard error; CI: confidence intervals; VDD: vertical defect depth; Estimate: β coefficient estimate. *p < 0.05. Statistically significant associations are depicted in bold.

**TABLE 1B:** Multiple linear regression analysis for vertical defect depth as the dependent variable with FOXP3, NRP‐1, IL‐10, TGF‐β1 and IL‐35 protein levels.

For VDD	Estimate (SE)	95% CI	*p*
FOXP3	0.04 (0.01)	[0.01; 0.07]	0.01*
NRP‐1	−0.40 (0.13)	[−0.66; −0.14]	< 0.01*
IL‐10	0.00 (0.00)	[0.00; 0.00]	0.44
TGF‐β1	−0.09 (0.08)	[−0.01; 0.00]	0.26
IL‐35	0.09 (0.14)	[−0.19; 0.37]	0.52

*Note*: All implants were considered for analysis (PI = 23, PI‐X = 23, H = 20). The regression models were adjusted for age and smoking status as covariates. BOP: bleeding on probing; OR: odds ratio; SE: standard error; CI: confidence intervals; VDD: vertical defect depth; Estimate: β coefficient estimate. *p < 0.05. Statistically significant associations are depicted in bold.

Apart from that, the protein levels of TGF‐β1 also showed a statistically significant inverse correlation with PD (*r*_*s*_ = −0.70, *p* < 0.001) (Figure 6D), but were positively associated with BOP (OR = 1.0[0.00], *p* < 0.001) (Table 1A). On the other hand, IL‐35 showed a significant direct correlation with PD (*r*_*s*_ = 0.53, *p* < 0.001) (Figure 6E) and was significantly associated with BOP presence (OR = 1.86[0.10], *p* < 0.001) (Table 1A). IL‐10 levels showed no associations with any of the clinical parameters.

## 4 Discussion

Altogether, our results confirm an altered Tregs profile and immune‐regulatory activity in human PI lesions. Specifically, *FOXP3* expression was significantly up‐regulated during PI compared to healthy tissues and implants belonging to the PI‐X group, whereas *NRP1* expression was consistently down‐regulated in the PI‐affected samples, suggesting an active but differentially impaired regulatory response. These findings were confirmed by the immunohistochemical analysis, revealing a higher number of FOXP3^+^ Tregs in PI and PI‐X tissues alongside a markedly reduced NRP‐1‐positive area, particularly when compared to the robust epithelial localisation observed in peri‐implant healthy tissues. Moreover, while IL‐10 levels remained relatively unaltered against PI, TGF‐β1 and IL‐35 expressions were significantly down‐regulated and up‐regulated, respectively, in the PI and PI‐X groups, suggesting the arrest of specific Tregs cytokine production pathways. Notably, the correlation analysis linked these molecular alterations with PI clinical severity, showing inverse correlations between NRP‐1 and TGF‐β1 with PD and VDD, while FOXP3 and IL‐35 exhibited a direct association with PD and BOP. Collectively, these findings suggest that Tregs‐compromised phenotype, particularly the alteration of FOXP3 and NRP‐1 expression, coupled with elevated IL‐35 and decreased TGF‐β1 levels, may be associated with the pathogenesis of PI.

PI lesions are characterised by the presence of a large infiltrate of diverse immune cells, among which T cells have been recurrently detected. One study reported that CD45^+^CD3^+^ T cells represented 22% of PI lesions infiltrating cells, with CD4^+^T helper cells comprising 41% of them (Ginesin et al. 2023). Similarly, CD3^+^ T cells detected within the peri‐implantitis connective tissue compartment represented 21% of infiltrated cells (6.87% ± 4.42% positive area, 4672 ± 5340 cells), also being significantly more than those detected in periodontitis‐affected tissues (Carcuac and Berglundh 2014). Consistently, the relative abundance of CD4^+^ T cells (*p* < 0.05) and specifically Tregs (*p* < 0.01) compartments were significantly larger in comparison with those present in periodontitis lesions (Oh et al. 2024). In fact, CD3^+^ T lymphocytes have been recently determined as the major immune cell population within the PI mucosa, with an average frequency of 57.6% ± 11.2% detected by flow cytometry (Malmqvist et al. 2024). Although the T‐cell subpopulation has been depicted as a common and significant portion of the PI immune profile, an in‐depth characterisation of their different subsets and phenotypes, including Tregs, remains vastly unexplored.

FOXP3, a transcription factor characteristically expressed in Tregs, is indispensable for their development and maintenance of their immunoregulatory/immunosuppressive phenotype (Ono 2020). As a master regulator, FOXP3 orchestrates the expression of genes essential for the suppressive activity of Tregs, including the production of immunoregulatory cytokines such as IL‐10, TGF‐β1 and IL‐35 (Maynard et al. 2007; Collison et al. 2007; Cafferata et al. 2020; Choi et al. 2020). However, during inflammatory conditions, abundant in pro‐inflammatory cytokines such as IL‐6 as observed during periodontitis and PI (Rojas et al. 2021; Tsukasaki et al. 2018; Chen et al. 2019), Tregs can lose their typical regulatory phenotype and acquire a pro‐inflammatory and osteolytic profile, instead. In this context, FOXP3 up‐regulation may not necessarily indicate effective suppression but rather a compensatory or unstable regulatory response. Indeed, Treg instability under inflammatory pressure has been reported, where FOXP3 expression persists transiently despite functional impairment (Min 2017; Zhou et al. 2009). Likewise, the down‐regulation of FOXP3 without the up‐regulation of Treg‐associated cytokine expression detected in PI lesions in the present study can be, at least in part, attributed to the dysregulated pro‐inflammatory microenvironment and, consequently, PI clinical severity. This suggests that while an increased FOXP3 expression may be associated with a response attempting to regulate active inflammation, it does not equate to functional suppression. Therefore, its subsequent decrease in the PI‐X group could reflect an impaired and ineffective regulatory function as the condition leads to implant failure.

NRP‐1 is expressed on highly activated CD4^+^ effector T cells and Tregs, where it contributes to immune homeostasis and self‐tolerance (Abberger et al. 2021). Notably, in the context of PI, the observed reduction of NRP‐1 expression may impair the function and stability of Tregs, leading to an exacerbated inflammatory response associated with disease pathogenesis. In fact, NRP‐1 silencing in Tregs significantly inhibits both their direct suppressive capacity over T effector cells and osteoclast differentiation and activity (Jung et al. 2020; Chen et al. 2019). Interestingly, Tregs co‐expressing NRP‐1 and FOXP3, representing ⁓45% of the CD4^+^CD25^+^ Tregs compartment, express significantly higher levels of FOXP3 than their NRP‐1^−^ counterparts (Battaglia et al. 2008). Otherwise, inflammatory conditions can negatively regulate the NRP‐1/Foxo3a axis, inducing Treg phenotype instability and promoting pro‐inflammatory osteolytic responses via the induction of Th17‐associated factors such as TNF, IL‐17A, M‐CSF and RANKL, ultimately leading to an increased osteoclastogenic burden (Chen et al. 2019; Taki et al. 2005). Thus, consistent with the marked decrease in NRP‐1 expression and its association with higher odds of BOP and VDD, NRP‐1^+^ cells may be down‐regulated during the PI inflammatory process, potentially reflecting an altered Treg regulatory mechanism that promotes peri‐implant tissue destruction and implant loss.

TGF‐β1 has high affinity for NRP‐1 binding on Tregs, while NRP‐1^−^ cells have minimal ability to bind and activate the latent form of TGF‐β1 (Battaglia et al. 2008; Glinka and Prud'homme 2008). Indeed, the co‐incubation of NRP‐1^−^ conventional T cells with soluble NRP‐1‐Fc resulted in the activation of TGF‐β1 and the acquisition of an immunosuppressive phenotype (Glinka and Prud'homme 2008), suggesting that TGF‐β1/NRP‐1 signalling is important for Tregs activity. Furthermore, TGF‐β1 overexpression has been detected within PI‐affected tissues and surrounding failing implants (Berryman et al. 2020; Cornelini et al. 2003), whereas some studies have shown that TGF‐β1 inhibition promotes osteoblast differentiation (Takeuchi et al. 2010), bone formation (Filvaroff et al. 1999) and early implant osseointegration (Cirera et al. 2020). Certainly, chronic inflammation and fibrosis can lead to persistently high levels of active TGF‐β1 (Ehnert et al. 2010; Insua et al. 2024), which can promote RANKL production in osteoblasts in vitro and favour bone resorption (Ehnert et al. 2010). Therefore, we can hypothesise that during PI, TGF‐β1‐dependent regulatory pathways could be compromised because of NRP‐1 down‐regulation while directly enhancing bone resorption.

IL‐35 is a highly immunosuppressive cytokine induced in response to overwhelming inflammatory stimuli, unlike IL‐10 or TGF‐β1 which are considered housekeeping anti‐inflammatory cytokines (Li et al. 2012). Its immune‐regulatory and osteoprotective roles during periodontitis are well documented. In experimental periodontitis, IL‐35 administration suppressed Th17 cells and significantly inhibited bone resorption (Cafferata et al. 2019, 2020). However, IL‐35 overexpression has also been associated with progressive inflammation severity (Zhang et al. 2015), while IL‐35 knock‐out increased T‐cell activity and animal mortality in experimental encephalomyelitis (Tirotta et al. 2013), suggesting that IL‐35 is up‐regulated during chronic inflammation in an effort to regulate it. Although IL‐35 overexpression has been associated with periodontitis and its progression (Mitani et al. 2015; Jin et al. 2017), this is, to our knowledge, the first report evaluating IL‐35 expression in PI lesions and crevicular fluid, as well as its association with PI clinical severity. Taken together, these results may reflect a reactive IL‐35‐driven immune‐modulatory response that may be insufficient to arrest the pro‐inflammatory challenge during PI.

The imbalance between FOXP3 and NRP‐1 expression, coupled with decreased TGF‐β1 and elevated IL‐35 levels, suggests a dysfunctional Treg response that fails to arrest chronic inflammation and may contribute to peri‐implant tissue destruction during PI. From a preventive perspective, early detection of immune dysregulation markers—such as altered NRP‐1 expression or IL‐35 levels—could aid in identifying patients at higher risk for PI progression, thus enhancing diagnosis and disease severity monitoring. Furthermore, therapeutic strategies aimed at restoring Treg function, stabilising FOXP3 expression or modulating NRP‐1 and TGF‐β1 signalling could offer novel immunomodulatory approaches to complement conventional mechanical and antimicrobial PI therapies (Cafferata, Monasterio, et al. 2021; Cafferata, Castro‐Saavedra, et al. 2021). For instance, targeted delivery of NRP‐1 agonists may contribute to the re‐establishment of peri‐implant immune homeostasis by promoting both Treg immune‐modulatory phenotype recovery/maintenance and Treg‐mediated alternative macrophage (M2) inflammation resolution (Sharma et al. 2020). Ultimately, understanding and leveraging the immune dysregulation in PI may pave the way towards more personalised, immune‐targeted therapies that improve PI clinical outcomes and implant longevity.

This study provides novel insights into the immune‐regulatory milieu of PI by integrating histological, molecular and clinical data focused on Treg‐associated markers. A key strength is the combined analysis of both transcriptional and protein expression, allowing a multifaceted characterisation of the Treg profile in peri‐implant tissues. To our knowledge, this is the first analysis of IL‐35 and NRP‐1 expression in human PI lesions and crevicular fluid, contributing valuable data to the largely unexplored field of Treg dynamics in PI. However, several limitations should be acknowledged. Our retrospective design limits causal inferences and precludes the direct assessment of disease progression. Associations between molecular and clinical parameters, while suggestive, cannot establish directionality or mechanistic causation. Moreover, the absence of flow cytometry or single‐cell analyses restricts precise Treg subset identification or functional assessment. Furthermore, the modest sample size and potential inter‐individual variability may affect the generalisability of the results. An additional consideration is the choice of healthy controls, which were obtained during second‐stage implant surgery and thus do not perfectly reflect the oral environmental conditions that healthy peri‐implant tissues are usually exposed to. While this may represent a biologically earlier timepoint compared to tissues surrounding functionally loaded implants, ethical limitations preclude the harvesting of soft tissues from healthy functioning implants. Indeed, although unloaded, these tissues were in contact with the cover screw and therefore not entirely isolated from implant–material interaction, thus at least partially emulating the healthy peri‐implant tissue immune response, as reported, for instance, when comparing neutrophil activity (Park et al. 2024). Despite these limitations, the observed associations between immunological alterations and PI clinical signs offer a valuable foundation for future ethically sound, longitudinal and mechanistic studies.

## 5 Conclusions

PI is associated with an altered Treg profile accompanied by an imbalanced immune‐regulatory cytokine milieu. The elevated FOXP3 expression coupled with the marked reduction in NRP‐1 in PI lesions, as well as the direct IL‐35 and inverse TGF‐β1 correlations with PI clinical parameters, suggest that although a regulatory response is present, it may be functionally impaired in the context of disease and that these molecular alterations are associated with PI osteolytic inflammation and clinical signs of severity.

## Author Contributions

**Emilio A. Cafferata:** conceptualization, data curation, funding acquisition, investigation, methodology, statistical data analysis, project administration, visualisation, writing original draft, review and editing of the final manuscript. **Ausra Ramanauskaite:** investigation, writing original draft, review and editing of the final manuscript. **Puria Parvini:** sample collection, review and editing of final manuscript. **Clemens Raabe:** data curation, investigation, visualisation, review and editing of final manuscript. **Eva Dohle and Shahram Ghanaati:** laboratory resources, methodology, final manuscript review. **Frank Schwarz:** funding acquisition, project administration, supervision, review and editing of final manuscript.

## Ethics Statement

The study was approved by the local ethics committee (registration number: 2022–652) and executed in accordance with the Helsinki Declaration, as revised in 2013.

## Conflicts of Interest

The authors declare no conflicts of interest.

## Supporting information


**Table S1:** Demographic and clinical parameters of included implants/patients.
**Table S2:**. List of primers used during RT‐qPCR analysis.

## Data Availability

The data that support the findings of this study are available from the corresponding author upon reasonable request.
