# Supplementary material for: Impaired Treg‐Mediated Immune Regulation in Peri‐Implantitis Lesions and Implant Loss: Insights From Histological and Molecular Analyses
Source: J Clin Periodontol. 2025 Aug 26;52(12):1779–90. doi: 10.1111/jcpe.70026 (PMC12605793; doi:10.1111/jcpe.70026)
Supplement: Supplementary file 1 — Table S1: Demographic and clinical parameters of included implants/patients. Table S2:. List of primers used during RT‐qPCR analysis. [file JCPE-52-1779-s001.docx]

**Supplementary Material**

**2** | **Materials and methods**

*2.1* | *Study design.*

The present study has a retrospective design, based on the assessment of clinical records and matching preserved peri-implant tissue samples from the archive of the Department of Oral Surgery and Implantology, Carolinum, Goethe University, Frankfurt am Main. The archived clinical parameters and histological data analysis is subsequently detailed.

*2.2* | *Participants’ selection.*

Clinical records and previously preserved peri-implant tissue samples from a total of 66 patients, who attended the Department of Oral Surgery and Implantology, Carolinum, Goethe University, Frankfurt am Main from 2022 to 2024 were recovered. The study protocol was in accordance with the Helsinki Declaration, as revised in 2013 and approved by the local ethics committee (registration number: 2022–652). From them, 46 had a clinical diagnosis of PI, of which 23 (n = 23 implants) underwent PI surgical therapy (PI group), and 23 (n = 23 implants) underwent explantation (PI-X group). Twenty patients presented exclusively healthy implants (i.e., without signs of progressive bone loss and no bleeding on probing) and served as a negative control (PI-H group).

PI diagnosis was based on the combination of the following criteria: bleeding on probing (BOP) and/or suppuration (SUPP), probing depth (PD) ≥ 6 mm, and radiographic marginal bone loss (MBL) ≥3 mm (Berglundh et al., 2018). In addition, patients had to be at least 18 years old at the time of diagnosis, present more than 2 mm of keratinized mucosa at the affected implant, and have an adequate oral hygiene as evidenced by a plaque index < 1 (Löe., 1967), to be included. Explantation was indicated when PI-affected implants exhibited radiographic bone loss equal or major to 2/3 of the implant length and/or mobility. Before PI or explantation surgical therapy, the peri-implant clinical parameters of the included implant were recorded at six sites per implant, including: BOP; PD and vertical defect depth (VDD; taken during surgery-from the crest to deepest site of the defect). In the case of the H group, the clinical parameters, taken 1-3 months controls after loading, were recovered.

In order to be included, samples must have been harvested during: Surgical treatment of PI (PI group) or explantation (PI-X group) due to unresolving progressive bone loss and mobility, or second stage surgery for implant platform revealing (Healthy [H] group).

Samples were excluded if, at the time of surgery, the following were reported: oral mucosal lesions/pathologies, autoimmune and/or inflammatory disorders (e.g. uncontrolled diabetes mellitus [HbA1c > 7] or untreated periodontitis), antibiotic or corticosteroid/immunosuppresant therapy within 3 months of surgery, pregnancy/lactation and poor oral hygiene.

Patients with untreated periodontitis were excluded to minimize systemic and local inflammatory confounding effects on peri-implant tissue immune profiles (Ginesin et al., 2023, Rocuzzo et al., 2012)

*2.3* | *Peri-implantitis reconstructive* *surgical procedure and peri-implant tissue sample obtention*

All PI group patients received pre-operative supra-mucosal implant cleaning and were treated through a standardized surgical protocol, as previously described (Schwarz et al., 2017). Otherwise, failing implants from the PI-X group were not subjected to pre-operative supra-mucosal cleaning. In brief, following local anaesthesia (articaine, 1:200.000), buccal and lingual mucoperiosteal flaps were elevated to expose the peri-implant defect. Granulation tissues were removed and collected from the respective defect area using conventional plastic curettes (Straumann Dental Implant System; Institut Straumann AG, Basel, Switzerland). Then, in the PI group, implant surfaces were decontaminated using a rotating titanium brush (Korea Co., Ltd., Gyeonggi-do, Korea) under copious saline irrigation. Finally, intrabony defects were filled with collagen-coated xenograft (Bio-Oss® Collagen; Geistlich) and covered with a native collagen membrane (BioGide; Geistlich). Otherwise, in the PI-X group implant explantation surgery was performed using a minimally invasive reverse torque to reduce trauma to the surrounding bone and soft tissues. If necessary, a trephine bur was used to facilitate implant removal. The surgical site was thoroughly debrided, and in cases planed for 2^nd^ implantation, an alveolar ridge preservation with a collagen-coated xenograft (Bio-Oss® Collagen; Geistlich) was performed. The mucoperiosteal flaps were repositioned and sutured with non-resorbable sutures, which were removed after 10 days. Complementarily, healthy samples in the H group obtained from patients who underwent 2^nd^ stage implant uncovering surgery were used as controls. All surgical procedures were conducted by two experienced and calibrated surgeons. (A.R. and P.P.)

In the PI and PI-X groups, granulation tissue was carefully curetted directly from the implant surface and defect area. In the H group, soft tissue biopsies were collected from the mucosa directly overlying the implant cover screw using a circular biopsy punch. One biopsy was collected per patient. Each biopsy was then divided, using a sterile blade, into two portions: a small fragment (≈ 2-5 mg) destined for whole RNA extraction, and the rest preserved for immunohistochemistry: All samples –i.e. granulation tissue and healthy peri-implant tissue– were then immediately placed in:

- 10% Buffered formalin for 24-48 h, dehydrated in alcohol for immunohistochemistry, and saved at -80°C or (Galarraga-Vinueza., 2021),
- Ice cold TRIzol (Invitrogen, Thermofisher), and saved at -80°C for RT-qPCR (Cafferata et al., 2021).

Samples that were handled differently or for which no handling information was available were excluded from the analysis.

*2.4* | *Immunohistochemistry*

PI, PI-X and H tissue samples were fixated in 10% buffered formalin for 24-48 h. Afterwards, all samples were dehydrated using ascending grades of alcohol and xylol, and embedded in paraffin. Due to granulation tissue irregular morphology and lack of clear anatomical landmarks of, tissue orientation was not feasible for histological embedding. H samples were oriented prior to embedding by placing the portion of the tissue that had been in direct contact with the implant cover screw was placed facing downward in the embedding mold, in order to obtain coronal sections showing the outer epithelial surface toward the implant-facing interface. After deparaffinization, rehydration, and mounting of 10 μm thick tissue sections on positively charged glass slides, antigen unmasking was performed by heating the samples for 30 min in a buffer citrate solution at 96 °C. After quenching the activity of endogenous peroxidase with hydrogen peroxide block for 10 min at room temperature, non-specific binding sites were blocked with Ultra V Block solution for 5 min (Ultravision Quanto Detection System HRP, Thermofisher). Then, tissue sections were incubated overnight with the primary antibodies against FOXP3 (clone EPR22102-37) (dilution: 1:100, Abcam), or neuropilin (NRP)-1 (clone EPR3113) (dilution: 1:100, Abcam) in humid chambers at room temperature, followed by the application of the Ultravision Quanto Detection System HRP kit for their detection. 3,3'-diaminobenzidine (DAB) and 3-amino-9-ethylcarbazole (AEC) were used as chromogens for FOXP3 and NRP-1 revealing, respectively. Next, sections were counterstained with Mayer’s haematoxylin. As negative controls, an unspecific rabbit IgG antibody and the omission of primary antibodies were applied.

*2.5 |* *Immunohistochemical data analysis*

Digital images (original magnification x 100; BX53, Olympus, Hamburg, Germany) were obtained from each specimen and analysed using the ImageJ software. For each sample, a region of interest (ROI) consisting of four non-overlapping high-power fields were randomly selected per slide, ensuring each represented a distinct region of the tissue without repetition from areas with well-preserved tissue morphology. ROIs were defined to include cellular connective tissue zones while avoiding artifacts such as tears, folds, or edge effects.

For FOXP3^+^ cell quantification, images were converted to 8-bit grayscale, and a binary threshold was applied to isolate positively stained nuclei. The number of FOXP3^+^ cells was then counted and normalized to the total number of nuclei per field, yielding a percentage of FOXP3^+^ cells relative to the total cell population, as previously described (Cafferata et al., 2024, Ling et al., 2018).

For NRP-1^+^ area coverage quantification, membrane and cytoplasmic staining were considered. After background subtraction and manual removal of non-specific artifacts, the stained (dark) pixel area was calculated and expressed as a percentage of the total tissue area within the ROI, as previously described (Cafferata et al., 2024).

*2.6* | *RT-qPCR analysis*

RNA extraction: Each individual granulation tissue sample was homogenized and total cytoplasmic RNA was purified from each condition using ice-cold TRIzol Plus (Invitrogen), as previously described (Cafferata et al., 2020). Total RNA was resuspended in RNase/DNase-free water and kept at -80°C. Then, the first-strand cDNA was synthesized using a reverse transcription kit (SuperScript III; Invitrogen), following the manufacturer's instructions. Finally, 10 ng of cDNA was amplified using the corresponding primers and the KAPA SYBR Fast qPCR kit (KAPA Biosystems) in a real-time qPCR equipment (StepOnePlus; Applied Biosystems). Amplification reactions were conducted as follows: 95°C for 3 min, followed by 40 cycles of 95°C for 3 s, 60°C for 30 s, and finally a melting curve of 95°C for 15 s, 60°C for 1 min, and 95°C for 15 s, for the detection of unspecific product formation and false-positive amplification. Specific forward and reverse primers against *FOXP3*, *HELIOS*, *NRP1*, *IL10*, transforming growth factor (*TGF)B1* and *IL35B*, for the assessment of Tregs-associated activity were used (Table S1). *18S* rRNA expression levels were used as endogenous control.

*2.7* | *Peri-implant crevicular fluid sample analysis*

To complement peri-implant tissue analyses, peri-implant crevicular fluid samples—representing the extracellular inflammatory context—collected prior to surgery in the PI and PI-X groups, and during follow-up in the H group were analysed. For these, the target site was gently dried and isolated with cotton rolls and, if present, supragingival plaque was removed using a plastic curette while avoiding the marginal mucosa. Then, two paper strips were placed into the mesial and distal sites of the peri-implant sulcus of the implant affected or not by PI until reaching minimal resistance and kept in place for 30 seconds, as previously reported (Navarrete et al., 2023). Strips contaminated by saliva or blood were discarded. After the collection of crevicular fluid, the strips were placed in sterile vials containing 130 μL of 0.05% Tween-20 in PBS and centrifuged for 10 min at 1200 G at 4°C. The protein elution procedure was repeated twice. Then the levels of IL-10, TGF-β1 and IL-35 production were analyzed by ELISA, following the manufacturer’s recommendations. Data were obtained using an automated plate spectrophotometer.

*2.8* | *Data analysis*

For the relative quantification of FOXP3 positive cells, and NRP-1 positive covered area by immunohistochemistry, ROIs from ten non-consecutive, non-overlapping slides from each sample were randomly selected. Mean ± standard deviations of positive cells/surface percentage present in the PI or PI-X granulation or H tissues were calculated. RT-qPCR data were presented as relative fold-change by normalizing the transcription factor or cytokine mRNA expression to the *18S* rRNA expression using the 2^-ΔΔCt^ method. The ELISA results were calculated using a logistic equation of 4 parameters. Data were expressed as mean ± standard deviation and analysed in a cross-sectional manner. The normality of data distribution was determined with the Kolmogorov–Smirnov test. Due to the exploratory nature of the study, the differences between groups were analyzed using the Kruskal-Wallis test, and statistical differences were determined using the non-parametric Mann-Whitney U test.

The Spearman’s rank correlation was used to determine bivariate correlations between Treg-marker expression and PD, and directly visualized using scatter plots. Otherwise, multiple linear regression analysis was used to evaluate the association between Treg protein levels and VDD -bone resorption-. Additionally, a multiple logistic regression analysis was conducted to assess the association between Treg protein levels and the presence of BOP, considered as a binary outcome variable, with H implants assigned a value of 0 (no BOP) and implants in the PI and PI-X groups assigned a value of 1 (if BOP was present) or 0. To account for potential confounders, age (as a continuous variable) and smoking status (current smoker vs. non-smoker) were included in the models as covariates. The sample size for the present study was determined considering a 5% significance level (α = 0.05), an 80% statistical power (1 − β = 0.80), and an effect size f = 0.65 (determined with a pilot comparing IL-35 protein levels between groups), resulting in a minimal n = 20 for detecting differences between groups with the Kruskal-Wallis test. Data were statistically analyzed using Microsoft Excel and the Jamovi v.2.3 software programs. The level of significance was set at *p* < 0.05.

**Table S1.** Demographic and clinical parameters of included implants/patients.

|  | **Peri-implant health** (H, n = 20) | **Peri-implantitis**  (PI, n = 23) | **Peri-implantitis explanted** (PI-X, n = 23) |
| --- | --- | --- | --- |
| Age  (mean ± SD) | 56.01 ± 11.56 | 62.22 ± 8.95 | 66.84 ± 11. 70 |
| Sex  (n, %) | 11 (55.0 %) | 15 (65.22 %) | 13 (56.52 %) |
| Smokers  (n, %) | 4 (20.0 %) | 7 (30.43 %) | 7 (30.43 %) |
|  | | | |
| PD  (mean ± SD) | 1.83 ± 0.40 | 4.25 ± 1.48 | 6.08 ± 1.26 |
| BOP  (mean ± SD) | 0.0 ± 0.0 | 0.85 ± 0.37 | 0.94 ± 0.22 |
| VDD  (mean ± SD) | - | 4.05 ± 0.93 | 4.95 ± 1.15 |

**Table S2**. List of primers used during RT-qPCR analysis

| **Gene target** | **Forward sequence** | **Reverse sequence** |
| --- | --- | --- |
| *FOXP3* | ACAGTCTCTGGAGCAGCAGC | CCACAGATGAAGCCTTGGTC |
| *HELIOS* | AGCTTTCACCCGAAAGGGAG | CTCATCTTCACGGCTCAGGG |
| *NRP1* | TCTCTCCACGCGATTCATCA | GCCTGGTCGTCATCACATTC |
| *IL10* | GGAGGTGATGCCCCAAGCTGA | AATCGATGACAGCGCCGTAGC |
| *TGFB1* | GGACACCAACTATTGCTTCAG | GTCCAGGCTCCAAATGTAGG |
| *IL35B* (*EBI3*) | CACTGAAGTACTGGATCCGT | GGAGACTCCAGTCACTCAGT |
| *18S* rRNA | CTCAACACGGGAAACCTCAC | CGCTCCACCAACTAAGAACG |

Reference

Galarraga-Vinueza, M. E, K. Obreja , C. Khoury, et al. 2021 . “Influence of Macrophage Polarization on the Effectiveness of Surgical Therapy of Peri-Implantitis”. International Journal of Implant Dentistry. 7, no. 1: 110. https://doi.org/10.1186/s40729-021-00391-2.
